# Role of Mitochondrial Stress Protein HSP60 in Diabetes-Induced Neuroinflammation

**DOI:** 10.1155/2020/8073516

**Published:** 2020-04-17

**Authors:** Donisha Shani Niharika Keembiya Liyanagamage, Ryan D. Martinus

**Affiliations:** School of Science, Division of Health, Engineering, Computing & Science, The University of Waikato, Hamilton, New Zealand

## Abstract

Diabetes mellitus is the most common metabolic disorder characterized by hyperglycemia and associated malfunctions of the metabolism of carbohydrates, proteins, and lipids. There is increasing evidence of a relationship between diabetes and vascular dementia. Interestingly, hyperglycemia-linked neuroinflammation in the central nervous system is considered to play a key role during vascular dementia in diabetic patients. However, the mechanisms responsible for the relationship between hyperglycemia and neuroinflammation is not clearly understood. Diabetes-induced alternations in the blood-brain barrier permit high glucose influx into the brain cells via glucose transporters and promote oxidative stress through overproduction of reactive oxygen species. Despite many studies demonstrating a link between oxidative stress and mitochondrial dysfunction, the relationship between mitochondrial dysfunction and neuron inflammation during hyperglycemia remains to be established. In this review, we will focus on diabetes-induced changes in the central nervous system and the role of mitochondrial heat shock protein 60 (HSP60) as an initiator of oxidative stress and potential modulator of neuroinflammation. We suggest that oxidative stress-mediated mitochondrial dysfunction stimulates the upregulation of mitochondrial heat shock protein 60 (HSP60) and ultimately initiates inflammatory pathways by activating pattern recognition receptors. HSP60 also could be a focal point in the development of a biomarker of neuroinflammation as HSP60 is known to be significantly elevated in diabetic patients. Interestingly, extracellular secretion of HSP60 via exosomes suggests that inflammation could spread to neighboring astrocytes by activating pattern recognition receptors of astrocytes via neuronal exosomes containing HSP60. A mechanism for linking neuron and astrocyte inflammation will provide new therapeutic approaches to modulate neuroinflammation and therefore potentially ameliorate the cognitive impairment in diabetic brains associated with vascular dementia.

## 1. Diabetes Mellitus

Diabetes mellitus is an endocrine disease characterized by hyperglycemia which occurs as a result of the inability of the pancreas to secrete insulin, defects in insulin action, or both [1]. Diabetes is classified into three main categories as type 1, type 2, and gestational diabetes based on the clinical manifestation [2]. Type 1 diabetes is characterized by hyperglycemia due to the cellular-mediated autoimmune destruction of pancreatic *β* cells which leads to the production of less insulin. In contrast, type 2 diabetes is described as the incapability of the body to effectively respond to insulin which leads to insulin resistance and relative insulin deficiency. Gestational diabetes is explained as any degree of glucose intolerance that is recognized during pregnancy which resolves on delivery of the placenta [3]. Hyperglycemia results in both microvascular and macrovascular complications which lead to long-term failure of various organs [4]. Oxidative stress-induced events are considered the most important unifying pathogenic factor that is responsible for the initiation and progression of diabetic complications [5]. Hyperglycemia-induced oxidative stress is characterized by the production of reactive oxygen species (ROS) via multiple pathways such as glucose autooxidation, increased metabolic flux of the polyol (sorbitol) pathway, increased production of advanced glycation end products (AGE), activation of protein kinase C, and increased flux of the hexosamine pathway [5].

## 2. Vascular Complications of Diabetes (Atherosclerosis)

Atherosclerosis is the formation of cholesterol plaque in the walls of arteries and leads to obstruction of normal blood flow [6]. Hyperglycemia is responsible for many alternations in vascular endothelial and smooth muscle tissues and initiates local inflammation in the vascular wall [7]. Injured vascular endothelial cells stimulate the adhesion of monocytes, and T lymphocytes infiltrate into the vascular intima through vascular walls. Subsequently, monocytes penetrate the subendothelial space, differentiate, and mature into macrophages that release cytokines. In diabetic patients, hyperglycemic condition results in the elevation of apolipoproteins in systemic circulation. High levels of low-density lipoproteins (LDL) facilitate the infiltration of LDL cholesterol molecules into subendothelial space and retention in the intima where it can be oxidized. Hyperglycemia also leads to the formation of several reactive oxygen species capable of promoting LDL oxidation [8]. Consequently, arterial macrophages can take up oxidized lipoproteins and stimulate the formation of foam cell which ultimately lead to atherogenesis [9]. Ritarwan and coworkers have successfully modeled the occurrence of atherosclerosis in streptozotocin-treated mice with increasing blood glucose and cholesterol level [10].

## 3. Diabetes-Induced Complications in the Central Nervous System

### 3.1. Diabetic Brain

The brain primarily utilizes glucose as a source of energy, which enters the brain through glucose transporters (GLUT-1) by crossing the blood-brain barrier [11]. The blood-brain barrier (BBB) is the protective gate of the central nervous system which prevents the entrance of potentially harmful substance. Endothelial cells connecting with tight junctions (TJ) construct the fundamental structure of the BBB. Pericytes and astrocytes contribute to the formation of basement membrane of the BBB [12]. Intercellular proteins such as claudin, occludin, and junctional adhesion molecules (JAMS) are responsible for producing the endothelial tight junctions which act as a filter for protein and cells across the vessel wall [13]. BBB regulates the transport of important molecules including glucose to maintain the brain homeostasis. Hyperglycemic condition is known to cause BBB impairments in diabetic patients which lead to the pathogenesis of various brain vascular complications [14]. In the diabetic patients, hyperglycemia facilitates a high respiration rate in pericytes and astrocytes which leads to high ROS production and oxidative stress [15]. Enhanced ROS production stimulates the upregulation of inflammatory cytokines and activation of the NF-*κ*B pathway which promote BBB leakages [16]. In addition, high concentration of ROS also inhibits the folding of gap junction proteins and disturbs astrocyte communication pathways [17]. Eventually, these impairments lead to inflammation-induced BBB opening and facilitate the entrance of high glucose influx into the central nervous system [17, 18].

### 3.2. Hyperglycemia-Induced ROS Production in the Neuron

The brain is one of the most vulnerable organs to oxidative stress [19]. Hyperglycemia-induced overproduction of ROS is considered as a key pathological factor in neuronal dysfunction which leads to cognitive impairments [20]. Prolong exposure to high glucose level stimulates various irregular glucose metabolic pathways [21]. In hyperglycemia, excess glucose generates metabolites in the polyol pathway and autooxidation of glucose which leads to the formation of advanced glycation end products [5]. In the polyol pathway, glucose is reduced to sorbitol, which is subsequently oxidized to fructose in the presence of aldose reductase [22]. These reactions oxidize NADPH to NADP^+^ as well as NADH from NAD^+^. Therefore, activation of the polyol pathway results in low concentration of NADPH and oxidized NAD^+^ which are important cofactors in redox reactions [22]. Elevated glucose concentration also undergoes nonenzymatic glycation reactions with primary amino acids and forms stable covalent products called advanced glycation end products (AGE). Subsequently, AGEs bind to a cell surface receptor known as receptor for AGE (RAGE) and induce excess production of ROS [23]. High concentration of ROS is considered to be hazardous for neuronal development and function. It initiates the misfolding of proteins in neuronal mitochondria [24]. Overproduction of ROS in the central nervous system also enhances tissue damage and disrupts neuronal regeneration [25].

### 3.3. Oxidative Stress-Induced Mitochondrial Dysfunction in the Neuron

The mitochondrion is the major organelle in the ATP production through electron transport chain and oxidative phosphorylation [26]. Neurons have high energy demands, and neuronal mitochondria supply constant energy for the adequate function of the neuron cells [27]. Brain neurons are mostly vulnerable to oxidative stress due to high consumption of oxygen, high concentration of membrane polyunsaturated fatty acids, and moderate antioxidant defense mechanisms [28]. Recent studies have demonstrated the critical role of the mitochondria in the progression of hyperglycemia-induced neuronal damage. Impairment of the mitochondrial function is a key feature of many neurodegenerative diseases [29]. Mitochondrial oxidative stress initiates a series of processes including mitochondrial DNA mutation, dysfunction in respiratory chain activity, detrimental changes in mitochondrial membranes, and defects in Ca^2+^ homeostasis [30]. *In vitro* diabetic neuropathy models have shown that excess ROS damages the mitochondrial electron transport chain, cellular proteins, lipids, and DNA and subsequently interferes with the normal mitochondrial function in dorsal root ganglion neurons [31]. Mitochondrial DNA is particularly susceptible to oxidative stress as the mitochondrial DNA lacks histone proteins and contains inefficient DNA repair systems [32]. However, mitochondria do contain a family of proteins (molecular stress proteins) which combat mitochondrial protein misfolding during oxidative stress.

### 3.4. Role of Molecular Stress Proteins during Mitochondrial Dysfunction

Mitochondrial stress is characterized by the accumulation of unfolded proteins which ultimately lead to impaired mitochondrial function [33]. It is evident that hyperglycemia-induced alternations in mitochondrial electron transport are a key causative factor for mitochondrial dysfunction [34]. Molecular stress proteins are a group of proteins which enable mitochondria to respond to stress-induced protein misfolding [35]. Heat shock protein 60 (HSP60) is one such molecular stress protein that is expressed in both prokaryotes and eukaryotic cells [36]. HSP60 is synthesized in the cytosol as a response of cellular stress and subsequently targeted the mitochondria, where it contributes to mitochondrial protein homeostasis [37]. HSP60 assists mitochondrial protein folding as well as denatured polypeptides in an ATP-dependent manner with the cochaperon HSP10 [38]. Recent studies have discovered that HSP60 knockdown decreases mitochondrial activity by increasing cell proliferation [39]. Afroz and coworkers have also revealed the interaction of HSP60 with mitochondrial proteins which promotes deregulation of mitochondrial function during infections [40]. Interestingly, HSP60 can also be translocated to the cell surface and secreted into the extracellular environment, where it can importantly function as an inflammatory regulator.

## 4. Immune Regulatory Action of Heat Shock Protein 60

### 4.1. Innate Immune System and CNS Inflammation

The immune system of the body consists of antigen-specific adaptive immune response as well as innate immune response [41]. The innate immune system is essentially protecting the body from viruses, bacteria, parasites, and other foreign particles by limiting their ability to spread and move throughout the body [42]. The innate immune system operates in the body mainly through pattern recognition receptors (PRRs). Pattern recognition receptors (PRRs) are proteins that enable to recognize molecules frequently found in pathogens which are called as pathogen-associated molecular patterns (PAMPs) or molecules released by damaged cells which are known as damage-associated molecular patterns (DAMPs) [43]. Despite PRR being found in the cytosol, cellular, and endosomal membranes as well as in an extracellular environment, secreted forms can be found in the bloodstream and interstitial fluids [44]. PRR can be divided into subfamilies such as Toll-like receptors (TLRs), C-type lectin receptors (CLRs), NOD-like receptors (NLRs), RIG-I-like receptors (RLRs), and AIM2-like receptor (ALR) [45]. These receptors activate innate immune responses by producing inflammatory cytokines, type I interferon, and other mediators. Among these families, the TLR families are well-characterized receptors comprising ten members (TLR1–TLR10) in human [46]. TLRs locate in the cell surface or in intracellular compartments as endosome, lysosome, ER and specific lipids, proteins, and nucleic acids [47]. Inflammasomes are also a group of pattern recognition receptors mainly NLRs that enable the activation of the secretion of inflammatory mediators [48]. It is evident that inflammasomes are necessary in the recognition of PAMPs or DAMPS by Toll-like receptors. Inflammasomes can also be activated by ROS, lysosomal damage, and cytosolic K+ flux [48]. Activation of PRR subsequently leads to inflammation which provides a broad spectrum of defense mechanisms against invasion and distribution of foreign pathogens, tissue injury, and many irritants. However, chronic and uncontrolled inflammation often leads to tissue damage through overzealous inflammatory responses.

Hyperglycemia results in persistent cellular stress in neurons which leads to chronic inflammatory changes. Hyperglycemia-induced reactive oxygen species and alterations in the redox equilibrium which produce oxidative stress play a vital role in the stimulation of immune cells in the diabetic brain [49]. Oxidative stress in the CNS is considered a critical step in stimulating CNS inflammatory pathways [50]. It is evident that overproduction of ROS enables the activation TLRs and inflammasomes in the CNS leading to the activation of inflammatory pathways. The activation of transcription factor, nuclear factor-kappa B (NF-*κ*B), has been identified as a key for neural dysfunction in many *in vivo* models ([51]; Wen et al., 2018). It also stimulates inflammation-induced disruption of synaptic signaling which leads to impairments in learning and memory in diabetic individuals.

### 4.2. HSP60 in Neuroinflammation

Neuroinflammation is a result of an innate immune response in the CNS. Astrocytes, microglia, and neurons play a vital role in maintaining tissue homeostasis and contribute to an inflammatory response through cytokine secretion [52]. Many recent studies have provided the link between mitochondrial stress and inflammation through HSP60 which can stimulate inflammatory mediators [53]. Numerous *in vivo* models have shown that HSP60 has both beneficial immune regulatory functions and harmful inflammatory properties. Earlier, it was recognized that HSP60 is an intracellular chaperon protein, but later, HSP60 has been identified as an important extracellular antigen in the human body [54]. Now, it is clear that HSP60 can act as an inflammatory danger signal in the innate immune responses [55]. Toll-like receptors are mostly considered a sensor for PAMs by playing an important role in the innate immune responses. Interestingly, reports have demonstrated that mammalian HSP60 can interact with TLRs by providing a nonpathogen-derived ligand for TLR [56]. Interaction of HSP60 and TLR stimulates inflammatory signaling cascade which ultimately leads to the production of proinflammatory mediators such as TNF-*α*, IL-1*β*, IL-6, and IL-8 [56]. Tian et al. have discovered that the extracellular HSP60 stimulates inflammation through activating and upregulating TLR-2 and TLR-4 in cardiomyocytes [57]. It is postulated that extracellular upregulation of HSP60 in neuron cells may act as a potential mediator for neuroinflammation. Rosenberger and coworkers have extensively studied the interaction of HSP60 with TLRs and impact on the brain cell with *in vivo* wild-type mouse model [58]. Their findings have revealed that intrathecal administration of HSP60 can mediate neuroinflammation in neuron and glial cells through Toll-like receptor 4 and 2 (TLR4/2). Rosenberger et al. have also discovered the predominant expression of HSP60 in neuronal cells over microglia and astrocytes in the brain [58]. However, interaction of the CNS neuronal HSP60 and the members of the TLR family remains controversial as some studies have suggested that brain neurons activate inflammation through TLR-3 rather than TLR-2 and TLR-4 [59].  Table 1 shows the expression of TLR family members in CNS cells (adapted from [60]).

Moreover, nucleotide-binding oligomerization domain leucine-rich repeat and pyrin domain-containing 3 (NLRP3) which is another family of pattern-recognizing receptors has been identified as a promising receptor for HSP60 [61]. It is observed that HSP60 can regulate endogenous IL-1*β* production by mitochondrial stress-induced activation of the NLRP3 inflammasome pathway in the microglia [62]. Later, Swaroop et al. have corroborated their early findings and revealed that downregulating HSP60 enables the reduction of IL-1*β* production and inflammation in Japanese encephalitis virus- (JEV-) infected mice [62]. These findings suggest a link between mitochondrial stress and neuroinflammation via HSP60.

### 4.3. Extracellular HSP60

Even though HSP60 is predominantly found in mitochondria, recent data suggest that it is also capable of being localized in the cytosol, nucleus, cell surface, intercellular vesicles, and extracellular environments (such as in blood circulation) [63]. HSP60 is secreted from cells into the extracellular environment through either endoplasmic reticulum and the Golgi apparatus or binding with exosomes and lipid channels [64]. When mitochondria are under stress, HSP60 is upregulated and transported into the cellular membrane and Golgi apparatus [63]. At the cell membrane, HSP60 internalizes through membrane lipid raft-mediated endocytosis into endosomes [65]. HSP60 can also encapsulate into exosomes through multivesicular bodies and be transported to neighboring cells [66]. In addition, free soluble HSP60 molecules can be translocated into the extracellular environment via Golgi vesicles [67]. Increased extracellular HSP60 expression in target tissues as well as body fluids such as blood, saliva, and urine has been documented in various inflammatory diseases such as cancer, diabetes, atherosclerosis, rheumatoid arthritis, insulitis, and neuroinflammatory diseases [54, 68]. Hence, free or exosomal bound HSP60 could be a promising biomarker for the diagnosis of inflammation in brain cells. Recent studies have discovered a clear link between extracellular HSP60 and immune responses of tissues. However, the mechanism of secretion of extracellular HSP60 is not clearly understood. A descriptive study on structural and functional comparison of intracellular HSP60 and extracellular HSP60 also needs to be established.

### 4.4. Neuron-Astrocyte Communication through Exosome in the CNS

Exosomes are known as a group of vesicles produced by the cells which are secreted to the extracellular body fluids such as blood, urine, saliva, and cerebrospinal fluid. Recent studies have revealed that exosomes play an important role in cell-to-cell communication in normal and pathological conditions [69]. Growing evidence also suggested that exosomes reflect the functional status of the cell from which it originates, and it can affect the functions of the cells which it interacts with. Exosomes permit transport of various proteins including targeting and fusion proteins, cytoplasmic enzymes, and molecular stress proteins. Proteins involved with signal transduction proteins are also known to be associated with exosomes [66]. Exosomes circulate throughout the body, and their concentration is elevated in some pathological conditions. In recent years, many studies have discovered that exosomes play a crucial role in cellular communication, nerve regeneration, synaptic function, and immune responses in the central nervous system. Latest finding has demonstrated that secretion of exosomes in the central nervous system depends on glutamatergic activity and calcium influx [70].

Interestingly, exosomes have also been shown to be involved in the pathogenesis of neuroinflammation. Exosome-mediated astrocyte-neuron communication has been shown to be critical for the survival of neurons [71]. Guitart et al. have discovered that astrocyte-derived exosomes enabled the transport of prion protein which is an important protective protein against oxidative stress to neurons [72]. Interestingly, Morel and coworkers have characterized the neuron-astrocyte communication pathway revealing that neuron-secreted microRNA-encapsulated exosomes can modulate GLT1 protein in astrocytes [73]. It is evident that astrocyte-derived exosomes can transport misfolded pathogenic proteins and miRNAs ultimately promote neuroinflammation [74]. Many novel investigations have identified that neuron-secreted exosomes are critical factors in spreading neuroinflammation and neurodegenerative diseases [75].

Recent findings of Pascua-Maestro et al. have shown that astrocyte-derived exosomes transport apolipoprotein D to neurons aiding neuronal survival ([76]). Emerging findings have uncovered that exosomes are involved with the transport of innate immune receptors such as Toll-like receptor (TLR) 4 and NOD-like receptor 3 (NLRP3) which are responsible for secreting inflammatory mediators which leads to neuroinflammation [77]. Astrocytes are also able to secrete proinflammatory cytokine-loaded exosomes which stimulate the inflammation in neurons [78].

The most recent studies have focused considerable attention on the role of exosomes and pathogenesis of diabetes. It is becoming evident that exosomes are involved in the pathogenesis of diabetic complications through stimulating inflammation, lowering GLUT4 as well as insulin receptors [34]. Interestingly, these exosomes can transport abnormal molecules and microRNA in diabetic patients. In addition, exosomes play a significant role in the treatments of cognitive impairments in diabetic patients. Nakano and coworkers have revealed that intracerebroventricular injection of bone marrow-derived mesenchymal stem cells was able to restore the cognitive impairments in diabetic mice [79]. Kalani et al. have also discovered the therapeutic efficacy of miR-146a-loaded brain endothelial cell-derived exosomes for the inhibition of gene expression of prion protein in diabetic db/db mice. These exosomes have achieved significant improvement in prion protein-induced cognitive impairments in diabetic mice [80].

We propose that neurons might be able to communicate with astrocytes via exosomal protein such as HSP60 during cellular stress condition like hyperglycemia. Remarkably, exosomes have become promising candidates as markers of inflammation in the central nervous system. Their ability to be transported through the blood-brain barrier could be exploited by measuring exosome concentration in cerebrospinal fluid and plasma as an indicator of brain inflammation. This feature of exosome suggests that measurements of exosomal HSP60 in diabetic patients could also be exploited to predict neuroinflammation in the diabetic brain. Therefore, exosomal HSP60 could be a focal point of developing a biomarker of neuroinflammation in diabetes.

### 4.5. Studies on the Expression of HSP60 in Peripheral Tissues

Elevated expression of the HSP60 level in diabetic patients is well documented with recent studies on saliva and serum evaluation of diabetic individuals [81]. Diabetes has been found to be associated with upregulation of HSP60 via ROS-induced mitochondrial dysfunction. Hall and Martinus have revealed that hyperglycemia and oxidative stress are responsible for elevating the expression of HSP60 in human HeLa cells [82]. Their findings have successfully demonstrated that three and seven days of exposure of HeLa cells to 100 mM glucose significantly increase the ROS production and expression of HSP60 compared to the control group. Moreover, this investigation has also suggested that upregulation of HSP60 is strongly related to ROS-mediated processes. Later, Martinus and Goldsbury have reported that human monocyte leukemia cells (THP-1) grown under hyperglycemic conditions (25 mM glucose) are able to release significant HSP60 amount into growth media than control cells grown under normoglycemic condition [83]. Furthermore, the conditioned media obtained from THP-1 cells were able to induce the secretion of TNF-*α* in human vascular endothelium cells.

## 5. Conclusion and Molecular Model Linking Mitochondrial Stress and Neuronal Inflammation

HSP60, a molecular stress protein predominantly found in the mitochondrial matrix, is known to be upregulated and secreted from cells during hyperglycemia-induced mitochondrial stress. This extracellular HSP60 is also known to stimulate inflammation in peripheral vascular environments (endothelial cells). To date, there are no reports of hyperglycemia-induced inflammation in the central nervous system through HSP60-activated inflammatory pathways.

**Figure 1:**
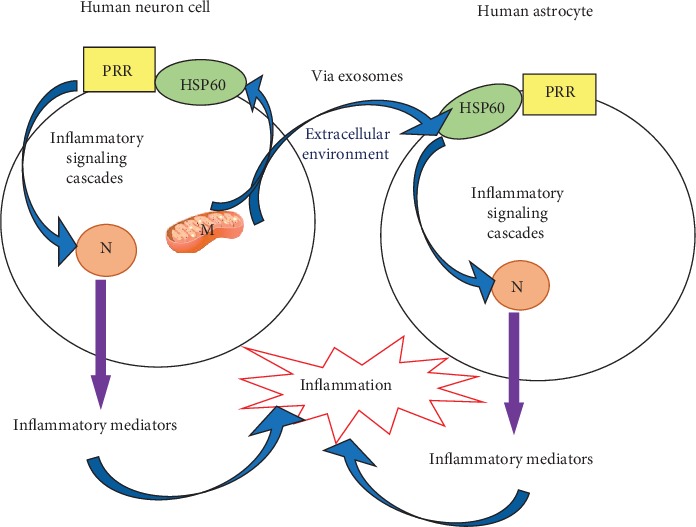
A model linking mitochondrial stress (via HSP60) to neuroinflammation. Hyperglycemia-induced overproduction of reactive oxygen species leads to mitochondrial dysfunction in neuron cells resulting in the upregulation of HSP60 expression. Excess HSP60 travels to the plasma membrane of neuron cell and binds with pattern recognition receptors. Subsequently, activated pattern recognition receptors initiate inflammatory signaling cascades, stimulate the production of inflammatory mediators, and ultimately lead to inflammation in neuron cell. Upregulated HSP60 in the neuron can also be secreted into the extracellular environment from neuronal mitochondria via exosomes. Afterward, extracellular exosomal HSP60 travels to neighboring astrocytes and binds with pattern recognition receptors on cell membrane of astrocytes, which in turn lead to triggering of an inflammatory response in astrocyte cells, thus contributing to neuroinflammation. PRR: pattern recognition receptors; HSP60: heat shock protein 60; N: nucleus; M: mitochondria.

We propose that hyperglycemia-induced upregulation of HSP60 expression in human neuronal cells may activate inflammatory pathways by stimulating Toll-like receptors which are located in the neuronal cell membrane to release proinflammatory cytokines. Furthermore, we hypothesize that upregulated HSP60 is secreted into the extracellular environment from neuronal mitochondria via exosomes. Moreover, we also suggest that extracellular exosomal HSP60 could travel to neighboring astrocytes and bind with Toll-like receptors on the cell membrane of astrocytes, which in turn would lead to triggering of an inflammatory response in astrocyte cells (Figure 1).

This potential molecular mechanism linking hyperglycemia to neuroinflammation in diabetes has implications for developing novel therapeutic approaches for the treatment of vascular dementia in diabetic patients.

## Figures and Tables

**Table 1:** Expression of TLR family members in CNS cells.

	Microglia	Neuron	Astrocyte	Oligodendrocyte
TLR 1	+	-	-	-
TLR 2	+	-	+	+
TLR 3	+	+	+	+
TLR 4	+	-	-	-
TLR 5	+	-	-	-
TLR 6	+	-	-	-
TLR 7	+	+	-	-
TLR 8	+	+	-	-
TLR 9	+	+	+	-

## References

[1] American Diabetes Association (2013). Diagnosis and classification of diabetes mellitus. *Diabetes Care*.

[2] American Diabetes Association (2018). Classification and diagnosis of diabetes. *Diabetes Care*.

[3] Solis-Herrera C., Triplitt C., Reasner C., DeFronzo R. A., Cersosimo E. (2018). Classification of diabetes mellitus. *Endotext*.

[4] Chawla A., Chawla R., Jaggi S. (2016). Microvasular and macrovascular complications in diabetes mellitus: distinct or continuum?. *Indian journal of endocrinology and metabolism*.

[5] Giacco F., Brownlee M. (2010). Oxidative stress and diabetic complications. *Circulation Research*.

[6] Kruth H. S. (2001). Lipoprotein cholesterol and atherosclerosis. *Current Molecular Medicine*.

[7] Rask-Madsen C., King G. L. (2013). Vascular complications of diabetes: mechanisms of injury and protective factors. *Cell Metabolism*.

[8] Linton M. F., Yancey P. G., Davies S. S. (2019). The role of lipids and lipoproteins in atherosclerosis. *Endotext*.

[9] Ganesan R., Henkels K. M., Wrenshall L. E. (2018). Oxidized LDL phagocytosis during foam cell formation in atherosclerotic plaques relies on a PLD2–CD36 functional interdependence. *Journal of Leukocyte Biology*.

[10] Ritarwan K., Lelo A., Pane Y. S., Nerdy N. (2018). Increasing atherosclerosis in streptozotocin-induced diabetes into four groups of mice. *Open access Macedonian journal of medical sciences*.

[11] Mergenthaler P., Lindauer U., Dienel G. A., Meisel A. (2013). Sugar for the brain: the role of glucose in physiological and pathological brain function. *Trends in Neurosciences*.

[12] Haddad-Tóvolli R., Dragano N. R. V., Ramalho A. F. S., Velloso L. A. (2017). Development and function of the blood-brain barrier in the context of metabolic control. *Frontiers in Neuroscience*.

[13] Günzel D., Yu A. S. (2013). Claudins and the modulation of tight junction permeability. *Physiological Reviews*.

[14] Barrett E. J., Liu Z., Khamaisi M. (2017). Diabetic microvascular disease: an endocrine society scientific statement. *The Journal of Clinical Endocrinology & Metabolism*.

[15] Li W., Roy Choudhury G., Winters A. (2018). Hyperglycemia alters astrocyte metabolism and inhibits astrocyte proliferation. *Aging and Disease*.

[16] Forrester S. J., Kikuchi D. S., Hernandes M. S., Xu Q., Griendling K. K. (2018). Reactive oxygen species in metabolic and inflammatory signaling. *Circulation Research*.

[17] Gandhi G. K., Ball K. K., Cruz N. F., Dienel G. A. (2010). Hyperglycaemia and diabetes impair gap junctional communication among astrocytes. *ASN neuro*.

[18] Prasad S., Sajja R. K., Naik P., Cucullo L. (2014). Diabetes mellitus and blood-brain barrier dysfunction: an overview. *Journal of pharmacovigilance*.

[19] Huang W.-J., Zhang X., Chen W.-W. (2016). Role of oxidative stress in Alzheimer's disease. *Biomedical Reports*.

[20] Lee H. R., Kong S. Y., Sung S. H., Kim H. J. (2019). DA-9801 and its saponins, dioscin and protodioscin, protect primary cortical neurons from hyperglycemia-induced neurotoxicity. *Journal of Functional Foods*.

[21] Ruud J., Steculorum S. M., Brüning J. C. (2017). Neuronal control of peripheral insulin sensitivity and glucose metabolism. *Nature Communications*.

[22] Yan L. J. (2018). Redox imbalance stress in diabetes mellitus: role of the polyol pathway. *Animal Models and Experimental Medicine*.

[23] Fournet M., Bonte F., Desmouliere A. (2018). Glycation damage: a possible hub for major pathophysiological disorders and aging. *Aging and Disease*.

[24] Salim S. (2017). Oxidative stress and the central nervous system. *Journal of Pharmacology and Experimental Therapeutics*.

[25] Krämer-Albers E. M. (2018). Exosomes deliver ROS for regeneration. *Nature Cell Biology*.

[26] Alberts B., Johnson A., Lewis J., Raff M., Roberts K., Walter P. (2002). *The Molecular Biology of the Cell*.

[27] Sheng Z. H. (2017). The interplay of axonal energy homeostasis and mitochondrial trafficking and anchoring. *Trends in Cell Biology*.

[28] Cenini G., Lloret A., Cascella R. (2019). Oxidative stress in neurodegenerative diseases: from a mitochondrial point of view. *Oxidative Medicine and Cellular Longevity*.

[29] Golpich M., Amini E., Mohamed Z., Azman Ali R., Mohamed Ibrahim N., Ahmadiani A. (2017). Mitochondrial dysfunction and biogenesis in neurodegenerative diseases: pathogenesis and treatment. *CNS Neuroscience & Therapeutics*.

[30] Guo C., Sun L., Chen X., Zhang D. (2013). Oxidative stress, mitochondrial damage and neurodegenerative diseases. *Neural Regeneration Research*.

[31] Sifuentes-Franco S., Pacheco-Moisés F. P., Rodríguez-Carrizalez A. D., Miranda-Díaz A. G. (2017). The role of oxidative stress, mitochondrial function, and autophagy in diabetic polyneuropathy. *Journal of Diabetes Research*.

[32] Santos R. X., Correia S. C., Zhu X. (2013). Mitochondrial DNA oxidative damage and repair in aging and Alzheimer's disease. *Antioxidants & Redox Signaling*.

[33] Valera-Alberni M., Canto C. (2018). Mitochondrial stress management: a dynamic journey. *Cell Stress*.

[34] Xiao Y., Zheng L., Zou X., Wang J., Zhong J., Zhong T. (2019). Extracellular vesicles in type 2 diabetes mellitus: key roles in pathogenesis, complications, and therapy. *Journal of Extracellular Vesicles*.

[35] Webster J. M., Darling A. L., Uversky V. N., Blair L. J. (2019). Small Heat Shock Proteins, Big Impact on Protein Aggregation in Neurodegenerative Disease. *Frontiers in Pharmacology*.

[36] Cappello F., Conway de Macario E., Marasà L., Zummo G., Macario A. J. (2008). Hsp60 expression, new locations, functions, and perspectives for cancer diagnosis and therapy. *Cancer Biology & Therapy*.

[37] Bavisotto C. C., Scalia F., Pitruzzella A., Górska-Ponikowska M., Marino C., Taglialatela G. (2019). Hsp 60 in modifications of nervous system homeostasis and neurodegeneration. *Heat Shock Protein 60 in Human Diseases and Disorders*.

[38] Yung H. W., Colleoni F., Dommett E. (2019). Noncanonical mitochondrial unfolded protein response impairs placental oxidative phosphorylation in early-onset preeclampsia. *Proceedings of the National Academy of Sciences*.

[39] Teng R., Liu Z., Tang H. (2019). HSP60 silencing promotes Warburg-like phenotypes and switches the mitochondrial function from ATP production to biosynthesis in ccRCC cells. *Redox Biology*.

[40] Afroz S., Brownlie R., Fodje M., van Drunen Littel-van den Hurk S. (2019). The bovine herpesvirus-1 major tegument protein, VP8, interacts with host HSP60 concomitant with deregulation of mitochondrial function. *Virus Research*.

[41] Chaplin D. D. (2010). Overview of the immune response. *Journal of Allergy and Clinical Immunology*.

[42] Nicholson L. B. (2016). The immune system. *Essays in Biochemistry*.

[43] Mogensen T. H. (2009). Pathogen recognition and inflammatory signaling in innate immune defenses. *Clinical Microbiology Reviews*.

[44] Amarante-Mendes G. P., Adjemian S., Branco L. M., Zanetti L. C., Weinlich R., Bortoluci K. R. (2018). Pattern recognition receptors and the host cell death molecular machinery. *Frontiers in Immunology*.

[45] Jang J. H., Shin H. W., Lee J. M., Lee H. W., Kim E. C., Park S. H. (2015). An overview of pathogen recognition receptors for innate immunity in dental pulp. *Mediators of Inflammation*.

[46] Barreiro L. B., Ben-Ali M., Quach H. (2009). Evolutionary dynamics of human Toll-like receptors and their different contributions to host defense. *PLoS genetics*.

[47] Kawasaki T., Kawai T. (2014). Toll-like receptor signaling pathways. *Frontiers in Immunology*.

[48] Swanson K. V., Deng M., Ting J. P. Y. (2019). The NLRP3 inflammasome: molecular activation and regulation to therapeutics. *Nature Reviews Immunology*.

[49] Nita M., Grzybowski A. (2016). The role of the reactive oxygen species and oxidative stress in the pathomechanism of the age-related ocular diseases and other pathologies of the anterior and posterior eye segments in adults. *Oxidative Medicine and Cellular Longevity*.

[50] Muriach M., Flores-Bellver M., Romero F. J., Barcia J. M. (2014). Diabetes and the brain: oxidative stress, inflammation, and autophagy. *Oxidative Medicine and Cellular Longevity*.

[51] Bathina S., Das U. N. (2018). Dysregulation of PI3K-Akt-mTOR pathway in brain of streptozotocin-induced type 2 diabetes mellitus in Wistar rats. *Lipids in Health and Disease*.

[52] Sochocka M., Diniz B. S., Leszek J. (2017). Inflammatory response in the CNS: friend or foe?. *Molecular Neurobiology*.

[53] Juwono J., Martinus R. D. (2016). Does Hsp60 Provide a Link between Mitochondrial Stress and Inflammation in Diabetes Mellitus?. *Journal of Diabetes Research*.

[54] Coelho V., Faria A. M. C. (2012). HSP60: issues and insights on its therapeutic use as an immunoregulatory agent. *Frontiers in Immunology*.

[55] Habich C., Burkart V. (2007). Interaction of heat shock protein 60 with innate immune cells. *Heat Shock Proteins: Potent Mediators of Inflammation and Immunity*.

[56] Vabulas R. M., Wagner H., Schild H. (2002). Heat shock proteins as ligands of toll-like receptors. *In Toll-Like Receptor Family Members and Their Ligands*.

[57] Tian J., Guo X., Liu X. M. (2013). Extracellular HSP60 induces inflammation through activating and up-regulating TLRs in cardiomyocytes. *Cardiovascular Research*.

[58] Rosenberger K., Dembny P., Derkow K. (2015). Intrathecal heat shock protein 60 mediates neurodegeneration and demyelination in the CNS through a TLR4-and MyD88-dependent pathway. *Molecular Neurodegeneration*.

[59] Chen C. Y., Shih Y. C., Hung Y. F., Hsueh Y. P. (2019). Beyond defense: regulation of neuronal morphogenesis and brain functions via Toll-like receptors. *Journal of Biomedical Science*.

[60] Hanke M. L., Kielian T. (2011). Toll-like receptors in health and disease in the brain: mechanisms and therapeutic potential. *Clinical Science*.

[61] Agostini L., Martinon F., Burns K., McDermott M. F., Hawkins P. N., Tschopp J. (2004). NALP3 Forms an IL-1β-Processing Inflammasome with Increased Activity in Muckle-Wells Autoinflammatory Disorder. *Immunity*.

[62] Swaroop S., Sengupta N., Suryawanshi A. R., Adlakha Y. K., Basu A. (2016). HSP60 plays a regulatory role in IL-1β-induced microglial inflammation via TLR4-p 38 MAPK axis. *Journal of Neuroinflammation*.

[63] Meng Q., Li B. X., Xiao X. (2018). Toward developing chemical modulators of Hsp60 as potential therapeutics. *Frontiers in Molecular Biosciences*.

[64] Nativel B., Planesse C., Gasque P., Da Silva C. R., Meihac O., Viranaïcken W. (2019). Biology of extracellular HSP60. *Chaperokine Activity of Heat Shock Proteins*.

[65] Henderson B., Martin A. C. (2014). Protein moonlighting: a new factor in biology and medicine. *Biochemical Society Transactions*.

[66] Rashed M. H., Bayraktar E., Helal G. K. (2017). Exosomes: from garbage bins to promising therapeutic targets. *International Journal of Molecular Sciences*.

[67] Campanella C., Bucchieri F., Merendino A. M. (2012). The odyssey of Hsp 60 from tumor cells to other destinations includes plasma membrane-associated stages and Golgi and exosomal protein-trafficking modalities. *PLoS ONE*.

[68] Taha E. A., Ono K., Eguchi T. (2019). Roles of extracellular HSPs as biomarkers in immune surveillance and immune evasion. *International Journal of Molecular Sciences*.

[69] Zagrean A. M., Hermann D. M., Opris I., Zagrean L., Popa-Wagner A. (2018). Multicellular crosstalk between exosomes and the neurovascular unit after cerebral ischemia. Therapeutic implications.. *Frontiers in Neuroscience*.

[70] Saeedi S., Israel S., Nagy C., Turecki G. (2019). The emerging role of exosomes in mental disorders. *Translational Psychiatry*.

[71] Levy E. (2017). Exosomes in the diseased brain: first insights from in vivo studies. *Frontiers in Neuroscience*.

[72] Guitart K., Loers G., Buck F., Bork U., Schachner M., Kleene R. (2016). Improvement of neuronal cell survival by astrocyte-derived exosomes under hypoxic and ischemic conditions depends on prion protein. *Glia*.

[73] Morel L., Regan M., Higashimori H. (2013). Neuronal exosomal miRNA-dependent translational regulation of astroglial glutamate transporter GLT1. *Journal of Biological Chemistry*.

[74] Gupta A., Pulliam L. (2014). Exosomes as mediators of neuroinflammation. *Journal of Neuroinflammation*.

[75] Vogel A., Upadhya R., Shetty A. K. (2018). Neural stem cell derived extracellular vesicles: attributes and prospects for treating neurodegenerative disorders. *eBioMedicine*.

[76] Pascua-Maestro R., González E., Lillo C., Ganfornina M. D., Falcón-Pérez J. M., Sanchez D. (2018). Extracellular vesicles secreted by astroglial cells transport apolipoprotein D to neurons and mediate neuronal survival upon oxidative stress. *Frontiers in Cellular Neuroscience*.

[77] Montesinos J., Alfonso-Loeches S., Guerri C. (2016). Impact of the innate immune response in the actions of ethanol on the central nervous system. *Alcoholism: Clinical and Experimental Research*.

[78] Bianco F., Pravettoni E., Colombo A. (2005). Astrocyte-derived ATP induces vesicle shedding and IL-1 beta release from microglia. *The Journal of Immunology*.

[79] Nakano M., Nagaishi K., Konari N. (2016). Bone marrow-derived mesenchymal stem cells improve diabetes-induced cognitive impairment by exosome transfer into damaged neurons and astrocytes. *Scientific Reports*.

[80] Kalani A., Chaturvedi P., Maldonado C. (2017). Dementia-like pathology in type-2 diabetes: a novel microRNA mechanism. *Molecular and Cellular Neuroscience*.

[81] Yuan J., Dunn P., Martinus R. D. (2011). Detection of Hsp60 in saliva and serum from type 2 diabetic and non-diabetic control subjects. *Cell Stress and Chaperones*.

[82] Hall L., Martinus R. D. (2013). Hyperglycaemia and oxidative stress upregulate HSP60 & HSP70 expression in HeLa cells. *SpringerPlus*.

[83] Martinus R. D., Goldsbury J. (2018). Endothelial TNF-α induction by Hsp60 secreted from THP-1 monocytes exposed to hyperglycaemic conditions. *Cell Stress and Chaperones*.

[84] Summ O., Evers S. (2013). Mechanism of action of indomethacin in indomethacin-responsive headaches. *Current Pain and Headache Reports*.

[85] Xiao T., Liang X., Liu H., Zhang F., Meng W., Hu F. (2020). Mitochondrial stress protein HSP60 regulates ER stress-induced hepatic lipogenesis. *Journal of Molecular Endocrinology*.

